# Supplementary material for: From sequence to enzyme mechanism using multi-label machine learning
Source: BMC Bioinformatics. 2014 May 19;15:150. doi: 10.1186/1471-2105-15-150 (PMC4229970; doi:10.1186/1471-2105-15-150)
Supplement: Additional file 2 — Java code of ml2db. Additional file ml2db_code.tar.gz contains the Java source code to run the multi-label machine learning experiments and save the results to database. The code’s Javadoc is included. [file 1471-2105-15-150-S2.zip › additional file 2/ml2db/utils/doc/uk/ac/standrews/utils/main/database/class-use/TableRow.html]

Uses of Class uk.ac.standrews.utils.main.database.TableRow


JavaScript is disabled on your browser.


- Overview
- Package
- Class
- Use
- Tree
- Deprecated
- Index
- Help

- Prev
- Next

- Frames
- No Frames

- All Classes

## Uses of Class uk.ac.standrews.utils.main.database.TableRow

- Packages that use TableRow

  | Package | Description |
  | --- | --- |
  |  |  |
  | --- | --- |
  | uk.ac.standrews.utils.main.database |  |
  | uk.ac.standrews.utils.test.database |  |
- - ### Uses of TableRow in uk.ac.standrews.utils.main.database

    Methods in uk.ac.standrews.utils.main.database that return TableRow

    | Modifier and Type | Method and Description |
    | --- | --- |
    |  |  |
    | --- | --- |
    | `TableRow` | TableReader.`getRowValues(java.sql.ResultSet resultSet, int rowNumber)` Get the nth row of a `ResultSet` |
    | `TableRow` | Table.`getTableRow()` |
    | `TableRow` | TableReader.`getTableRowWhereEqual(java.lang.String column, java.lang.String value)` Gets the \*first\* table row where the given column has exactly the given value |
    | `TableRow` | TableReader.`getTableRowWhereLike(java.lang.String column, java.lang.String value)` Gets the \*first\* table row where the given column has a value similar to the given value |

    Methods in uk.ac.standrews.utils.main.database that return types with arguments of type TableRow

    | Modifier and Type | Method and Description |
    | --- | --- |
    |  |  |
    | --- | --- |
    | `java.util.Vector<TableRow>` | TableReader.`getTableRowsWhereEqual(java.lang.String column, java.lang.String value)` Gets the table rows where the given column has exactly the given value |

    Methods in uk.ac.standrews.utils.main.database with parameters of type TableRow

    | Modifier and Type | Method and Description |
    | --- | --- |
    |  |  |
    | --- | --- |
    | `java.lang.String` | TableWriter.`getInsertSql(TableRow row)` Returns the sql "insert into" statement for the row |
    | `java.lang.Integer` | TableWriter.`insertReturnsKey(TableRow row)` Executes an sql 'insert into' operation. |
    | `java.lang.Integer` | TableWriter.`writeRowToTable(TableRow row)` Insert the row values into the appropriate database table |
  - ### Uses of TableRow in uk.ac.standrews.utils.test.database

    Methods in uk.ac.standrews.utils.test.database that return TableRow

    | Modifier and Type | Method and Description |
    | --- | --- |
    |  |  |
    | --- | --- |
    | `static TableRow` | TableRowTest.`getRowKo()` |
    | `static TableRow` | TableRowTest.`getRowOk()` |

- Overview
- Package
- Class
- Use
- Tree
- Deprecated
- Index
- Help

- Prev
- Next

- Frames
- No Frames

- All Classes
